# Oral anticoagulant therapy for patients with new-onset atrial fibrillation following acute myocardial infarction: A narrative review

**DOI:** 10.3389/fcvm.2022.1046298

**Published:** 2022-11-03

**Authors:** Shenglong Yu, Chenxi Li, Huizhuang Guo

**Affiliations:** ^1^Department of Cardiovascular, Panyu Central Hospital, Guangzhou, China; ^2^Medical Department, Queen Mary School, Nanchang University, Nanchang, China; ^3^Department of Radiology, Panyu Central Hospital, (Medical Imaging Institute of Panyu District), Guangzhou, China

**Keywords:** atrial fibrillation, new-onset, myocardial infarction, antithrombotic therapy, review

## Abstract

**Background:**

To evaluate the advantages and disadvantages of anticoagulant therapy and provide a piece of information on anti-thrombotic treatment strategies for patients with new-onset atrial fibrillation (NOAF) and acute myocardial infarction (AMI).

**Methods:**

Literature from PubMed and Google scholar were screened until August 2022. Studies assessing oral anticoagulant (OAC) treatments for NOAF in patients with AMI were evaluated for inclusion.

**Results:**

Three retrospective cohort studies were included. In the study performed by Madsen et al., patients with previously diagnosed AMI with or without NOAF were followed up for 5.8 years. About 38% of NOAF patients with anticoagulant therapies, which could reduce long-term mortality [adjusted hazard ratio (HR): 0.69; 95% confidence interval (CI): 0.47–1.00]. Hofer et al. performed a single-center cohort study containing 1,372 patients with AMI with an 8.6-year follow-up period. Dual anti-thrombotic therapy (DAT) did not show the effect on the survival in NOAF (adjusted HR: 0.97; 95% CI: 0.65–1.57), while triple antithrombotic therapy (TAT) could reduce long-term cardiovascular mortality (adjusted HR: 0.86; 95% CI: 0.45–0.92). Petersen et al. also did a cohort study with 1-year follow-up duration. It showed that anticoagulant therapies demonstrated positive results (HR: 0.78; 95% CI: 0.41–1.47).

**Conclusion:**

Recent studies have shown that anticoagulant therapy in AMI-NOAF patients can obviously reduce the mortality of AMI-NOAF patients, especially OAC therapy. Further clinical trials could confirm these findings.

## Introduction

Acute myocardial infarction (AMI) and atrial fibrillation (AF) are the most common cardiovascular disease and cardiac arrhythmia in the settings, respectively (1–3). The two diseases share common risk factors, (4) and the presence of either can lead to an increased risk of the other (5–7). Patients with AMI are frequently accompanied by new-onset atrial fibrillation (NOAF) based on multiple mechanisms, such as atrial ischemia, atrial stretch, severe autonomic activation, and hormonal activation (8–11). Recent studies have shown that NOAF following AMI (AMI-NOAF) is strongly correlated to the increased risks of stroke, recurrence of MI, and both short- and long-term mortality (12–16). Thus, the monitoring and treatment of these patients have been taken into serious consideration.

The antithrombotic therapy for AMI-NOAF patients is contradictory. For AMI, dual antiplatelet therapy (DAPT) with aspirin and a P2Y_12_ inhibitor is indicated to prevent stent thrombosis, (17) while in AF patients, oral anticoagulant (OAC) therapies including vitamin K antagonists (VKAs) or non-vitamin K oral anticoagulants (NOACs) are more effective in preventing stroke and other thromboembolic events (18, 19). However, triple therapy combining DAPT with an anticoagulant is usually associated with an increased rate of excessive bleeding, which limits the clinical application (20). During the past decades, large randomized clinical trials showed that using NOACs in patients with AF who had undergone percutaneous coronary intervention (PCI) may reduce the risk of bleeding compared to VKAs and DAPT without increasing the incidence of thrombotic events (21–23). Therefore, in this review, we evaluated the advantages and disadvantages of anticoagulant therapy and provided a piece of information on anti-thrombotic treatment strategies for patients with AMI-NOAF.

## Methods

Two investigators searched the electronic database until August 2022 independently. Relevant articles were screened from PubMed and Google scholar by using the following keywords: (AMI OR acute coronary syndrome) AND (atrial fibrillation) AND (non-vitamin K antagonist oral anticoagulants OR direct oral anticoagulants OR dabigatran OR rivaroxaban OR apixaban OR edoxaban OR VKAs OR warfarin). Studies were included if they assessed oral anticoagulant treatments (NOACs or VKAs) for NOAF in patients with AMI.

The corresponding searched results were recorded in Supplementary Table 1. After screening titles and abstracts of publications, two authors extracted data independently. Then, the full-text screening was performed to determine whether the literature met the inclusion criteria. Disagreements were resolved by discussing with the third researcher. The baseline information of each study was recorded, including the name of the first author, publication year, the types of anticoagulants, study design, baseline characteristics of the investigated population, and the study outcome.

## Results

**FIGURE 1:**
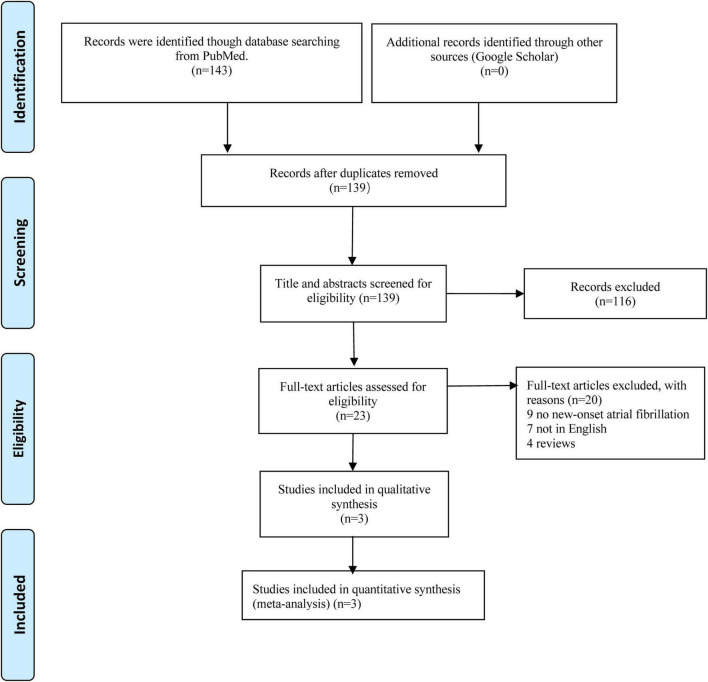
Diagram of retrieved study selection of this review.

Figure 1 shows the diagram of the retrieved study selection for this review. A total of 143 retrieved records were retrieved from the PubMed database. Firstly, titles and abstracts of all these records were screened, and then 120 studies were excluded according to the predetermined criteria. In the full-text screenings, we further excluded 20 studies because of the following reasons: (1) nine studies did not focus on new-onset AF, but the pre-existing AF, (2) seven studies were not written in English, and (3) four studies were reviews. Finally, three studies in total (24–26) (all of these studies were retrospective cohort studies) were included in this narrative review.

Table 1 shows the baseline characteristics of all included studies. Each study was published in 2021. The inclusion sample size for all included studies ranged from 1372 to 161266. A total of 170,257 patients in total with previously diagnosed AMI or acute coronary syndrome (ACS) and with or without NOAF. The following-up period was from the range of 1 to 8.6 years. The primary and second outcome results were also shown in Table 1.

**TABLE 1:** Summary of the relevant studies of this review.

References	Definition of NOAF	Study design	Follow-up period	Primary outcome results	Secondary outcome results
Madsen et al. (24)	A diagnosis of AF within 30 days after STEMI	Single-center, retrospective cohort study, only STEMI patients (*n* = 7,944)	5.8 years	Long-term all-cause mortality: STEMI with NOAF (*n* = 296) vs. without NOAF (*n* = 7,648): HR (95% CI) = 1.48 (1.20–1.82), *P* < 0.001; NOAF with OAC therapy (*n* = 113) vs. NOAF without OAC therapy (*n* = 183): HR (95% CI) = 0.69 (0.47–1.00), *P* = 0.049	Bleeding leading to hospitalization: STEMI with NOAF vs. STEMI without NOAF: HR (95% CI) = 1.36(1.00–1.85), *P* = 0.050; NOAF with OAC therapy vs. NOAF without OAC therapy: HR (95% CI) = 1.31(0.75–2.27), *P* = 0.34
Petersen et al. (25)	A diagnosis of AF during admission with ACS with no prior history of AF	Nationwide, retrospective cohort study, first-time admission with ACS (*n* = 161,266)	1 year	Ischemic stroke: ACS with a history of AF (*n* = 18,961), or NOAF (*n* = 6,427) vs. free of AF (*n* = 161,266): HR (95% CI) = 1.38 (1.22–1.56), 1.67 (1.38–2.01); OAC vs. antiplatelet therapy in patients with history of AF (*n* = 6,679), or NOAF (*n* = 2,331): HR (95% CI) = 0.87 (0.63–1.20), 0.78 (0.41–1.47)	All-cause mortality: With a history of AF, or with NOAF vs. without AF: HR (95% CI) = 1.25 (1.21–1.31), 1.52 (1.43–1.62); OAC vs. antiplatelet therapy in patients with history of AF, or with NOAF: HR (95% CI) = 0.75(0.68–0.84), 0.75(0.61–0.91); Bleeding: With a history of AF (6.9%), or with NOAF (5.7%) vs. without AF (3.6%): HR (95% CI) = 1.22(1.14–1.30), 1.28 (1.15–1.43); OAC vs. antiplatelet therapy in patients with history of AF, or with NOAF: HR (95% CI) = 1.18 (0.99–1.41), 1.11 (0.83–1.49)
Hofer et al. (26)	A new onset of atrial fibrillating impulses at the time of admission or during the hospitalization	Single-center, retrospective cohort study, AMI (STEMI or NSTEMI) patients	8.6 years	Long-term cardiovascular mortality: NOAF (*n* = 149) or pre-existing AF (*n* = 90) vs. free of AF (*n* = 1,133): HR (95% CI) = 1.45 (1.19–2.57), 0.70 (0.35–0.98); DAT (*n* = 21) or TAT therapy (*n* = 56) vs. DAPT only therapy (*n* = 32) in patients with NOAF: HR (95% CI) = 0.97 (0.65–1.57), 0.86 (0.45–0.92)	Fatal bleeding events: No difference between different treatment strategies: NOAF (*n* = 4): 2.6% vs. pre-existing AF (*n* = 2): 2.2%, *P* = 0.824

ACS, acute coronary syndrome; AF, atrial fibrillation; AMI, acute myocardial infarction; CI, confidence interval; DAPT, dual antiplatelet therapy; DAT, dual anti-thrombotic therapy: the combination of single anti-platelet therapy (aspirin or clopidogrel) and VKA; HR, hazard ratio; NOAF, new-onset atrial fibrillation; OAC, oral anticoagulant; STEMI: ST-segment elevation myocardial infarction; TAT: triple antithrombotic therapy: OAC plus DAPT; PCI, percutaneous coronary intervention; P2Y_12_-I, P2Y_12_ inhibitors.

Madsen et al. described a sizeable single-center cohort including 7,944 patients with ST-segment elevation myocardial infarction (STEMI) treated with PCI between 1999 and 2016 to investigate the prognostic implication of OAC therapy for AMI-NOAF, of which 75.2% were males (24). Among these patients with STEMI, 296 (3.7%) of them developed NOAF. It was reported that patients with NOAF were older, more frequently non-smoking women and often more likely to have cardiovascular comorbidities. NOAF can lead to increased long-term mortality [adjusted hazard ratio (HR) for STEMI with NOAF vs. without NOAF: 1.48; 95% confidence interval (CI): 1.20–1.82]. It can also increase the risk of bleeding leading to hospitalization (adjusted HR: 1.36; 95% CI: 1.00–1.85). About 38% of NOAF patients were treated with OAC therapy, which can decrease long-term mortality (adjusted HR: 0.69; 95% CI: 0.47–1.00).

Hofer et al. also performed a single-center retrospective cohort study containing 1,372 patients with AMI to observe the development of *de novo* AF. They found that in the acute phase of AMI, 149 (10.9%) patients developed NOAF. After 8.6 years of following up analysis showed that 30.5% of patients died because of cardiovascular diseases. These included 93 (62.4%) patients in the NOAF group. It was reported that NOAF has a strong correlation with long-term cardiovascular mortality (adjusted HR: 1.45; 95% CI: 1.19–2.57). Dual anti-thrombotic therapy (DAT) did not show the effect on the survival in NOAF (adjusted HR: 0.97; 95% CI: 0.76–1.21). While triple antithrombotic therapy (TAT) can reduce long-term cardiovascular mortality (adjusted HR: 0.86; 95% CI: 0.45–0.92). But a recent meta-analysis showed that DAT can reduce bleeding and has a similar effect on preventing AF with ACS or PCI (27). Thus, the optimal treatment regimen should be decided by experts in specific conditions.

Petersen et al. did a nationwide cohort study; 161,266 ACS patients were included. In patients with newly diagnosed AF, a high incidence of ischemic stroke was observed (HR: 1.38; 95% CI: 1.22–1.56). Also, compared to patients without AF, a higher mortality rate was in the NOAF group (HR: 1.52; 95% CI: 1.43–1.62). As for the recurrence of myocardial infarction, there was no significant difference was found in patients with firstly diagnosed AF (HR: 0.99; 95% CI: 0.91–1.07). And patients with NOAF also showed an increased rate of bleeding (HR: 1.28; 95% CI: 1.15–1.43). OAC treatment also showed positive results. It had the lowest incidence of ischemic stroke in both pre-existing AF (HR: 0.87; 95% CI: 0.63–1.20) and new-onset AF (HR: 0.78; 95% CI: 0.41–1.47), although the difference was not statistically significant.

There are also some limitations in these three studies. The study of Madsen et al. does not contain information on the cause of death. And some asymptomatic patients may have undiagnosed previous paroxysmal atrial fibrillation which may give rise to an underestimated incidence. Additionally, due to a lack of power, the analysis of OAC therapy in NEW-AF which may be a confounding factor was not included. The limitations of Hofer’s study were that there was no non-fatal data of ischemic stroke or bleeding complications, which may have an effect on the final results. Additionally, the follow-up visits of patients using DAT and TAT were not completed and the rates of AF episodes were not evaluated during the observation period. What’s more, this investigation was limited to VKAs. Because at that time, NOACs haven’t been approved by FDA and EMA. As for the limitations of Petersen’s study, first, this is an observational study, since the risk of residual confounding cannot be excluded, the assessment of antithrombotic therapy is challenging. Second, bleeding cannot be sorted by criteria. Third, the treatment regimens were drastically changed during the treatment period.

## Discussion

### Epidemiology of acute myocardial infarction-new-onset atrial fibrillation

Arrhythmia is not uncommon during the acute phase of AMI. The incidence of NOAF in patients with AMI varies among studies with a wide range from 2 to 21% (28–30). Approximately half of NOAF developed within 30 days of the onset of AMI (31). Notably, the onset of NOAF was not evenly distributed, as 30% of events occurred at the time or within 2 days after AMI, 16% during the intermediate stage of 3 to 30 days after AMI, while 54% occurred more than 30 days with gradually decreased during follow-up (31). Previous studies have shown that the incidence of NOAF after AMI ranges from 3.7 to 22.6% (3–7). Due to the loss of effective atrial contraction, increased ventricular rate, shortened ventricular diastolic time, irregular RR interval and other factors during AF, the decrease in ventricular filling and ejection, the decrease in coronary blood supply and the increase of myocardial oxygen consumption aggravates the degree of cardiac injury in patients with AMI. In addition, after the loss of normal atrial systolic function, atrial blood flow stagnation or turbulence can easily lead to thrombosis. These factors make patients with AMI more prone to hospital complications.

### Risk factors of acute myocardial infarction-new-onset atrial fibrillation

Atrial fibrillation shares a couple of risk factors with AMI, such as aging, hypertension, obesity, diabetes, alcohol consumption, and sleep-disordered breathing (4, 5, 18). Therefore, the two diseases may share similar pathophysiological pathways, and the co-occurrence of these two diseases seems not to be avoidable (4). The cause of AMI-NOAF is multifactorial with older age, female sex, hypertension, cardiogenic shock, and congestive heart failure have been identified as risk factors (32, 33). The conventional view holds that infarct size and severity contribute to the development of AF after AMI (34, 35). In contrast, a recent community cohort study of 3,220 people conducted by Jabre et al. found that AMI characteristics and indicators of severity, except anterior location, higher Killip levels, and lower left ventricular ejection fraction, are mostly irrelevant to the occurrence of NOAF (31). NOAF occurs in a short time after AMI. Atrial ischemia, atrial infarction, atrial dilatation and elevated intraatrial pressure may be the main causes in the early stage, while inflammation, oxidative stress and atrial remodeling involved in autonomic nerves may be the main causes in the later stage (8–11). The mechanism of NOAF after AMI is complex and has not been fully elucidated at present.

### Mechanisms of acute myocardial infarction-new-onset atrial fibrillation

Since coronary occlusion is the pathogenesis of AMI, the resulting further myocardial ischemia is considered the most critical mechanism for the onset of NOAF. A case-control study conducted by Alasady et al. (36) demonstrated that approximately half of the AMI-NOAF patients had a critical lesion in the sinoatrial nodal branch originating from the right coronary or left circumflex arteries, which was 25-times more than the patients free of AF. Therefore, the atrial branch affected by coronary artery disease is considered a strong predictor of AMI-NOAF. Hofer et al. (26) and Alasady et al. (36) found that patients with AMI-NOAF were significantly less likely to receive timely PCI and stent implantation compared to those free of AF, resulting in broader tissue damage and scar formation. The peak creatinine kinase value is a surrogate marker for evaluating the infarct size (37), while the N-terminal proB-type natriuretic peptide (NT-proBNP) level indicates cardiac strain. In the research mentioned above, both creatinine kinase and NT-proBNP were elevated significantly in the AMI-NOAF patients, suggesting that the tissue damage and overstretch as the results of myocardial ischemia may develop an extended electrical and structural remodeling of the heart, and trigger the onset of AF. In animal models, ligation of the atrial branch of the right coronary artery would result in isolated atrial ischemia. In the ischemia region, there is a significantly decreased conduction velocity of atrial cardiomyocytes, which may promote and stabilize reentry that maintains AF (38). Additionally, inflammation reaction may also relate to AF. Psychari et al. (39) showed that the level of IL-6 in AF patients is obviously higher than it in non-AF patients. Thus, patients with AMI may generate systemic inflammatory response which may be responsible for NOAF. Yoshizaki et al. (40) demonstrated that in patients with NOAF following AMI, the level of white blood cell and C-reaction protein are higher than in patients with no NOAF (41).

### Prognosis of acute myocardial infarction-new-onset atrial fibrillation

The general clinical characteristics of patients with NOAF after AMI are old age, low blood pressure, higher admission heart rate, higher Killip grade, more severe coronary artery disease and so on. Poor general condition directly affects the patient’s condition and increases the difficulty of treatment, which increases the risk of in-hospital heart failure, re-infarction, cerebral infarction and hemorrhage, resulting in poor short-term and long-term prognosis and increased mortality. The SPRINT trial compared the pre-thrombolytic era with the thrombolytic era, the 30-day and 1-year mortality rates of patients with AF after AMI in the pre-thrombolytic era were 27.6 and 42.5%, respectively, and the 30-day and 1-year mortality rates of patients with paroxysmal AF after MI in the thrombolytic era were 25.1 and 38.4%, respectively (42). Through further multivariate adjustment, it was found that the mortality of patients with AF after MI in the thrombolytic era was significantly lower than that in the pre-thrombolytic era. The incidence of NOAF after PCI in the OACIS study was 12.0% (7). A meta-analysis including 43 studies (278,854 participants) showed that among patients with AMI, the presence of AF would lead to at least a 40% higher mortality rate than those with sinus rhythm, while this poor prognosis persists regardless of the timing of AF onset (29). Notably, AMI-NOAF was still associated with an increased risk of death, even after adjusting for risk factors such as age, diabetes, hypertension, prior AMI, heart failure, and coronary revascularization status (29). Nevertheless, the prognosis of cardiovascular disease and death related to the first detection of AF in ACS remains to be further elucidated.

### Antithrombotic therapy for acute myocardial infarction-new-onset atrial fibrillation

Myocardial infarction is usually caused by a rupture of the plaque on the basis of a severe stenosis of the coronary artery, leading to thrombosis. AMI can initiate atherosclerotic plaques which are prone to rupture, owing to the high level of lipids and apoptotic cells, which leads to a fatty core and thin fibrous cap (43). Thus, thrombosis is formed and endothelial coverage is lost. This triggers two main pathways. One is coagulation activation, the other is platelet activation (44). Platelet recruitment is also related to two pathways. The first is dependent on the coagulation cascade. The second is associated with tissue factor release. AF can also lead to coagulation disorder through thrombogenesis by affecting the coagulation cascade (45). Thus, antithrombotic therapy is necessary for AMI-NOAF treatment.

Because there are plenty of pathways and factors in the coagulation process which is related to thrombosis formation. It is crucial to block any of them so that can people effectively decrease the risk of thrombosis formation. There are some examples. First, inhibiting platelet aggregation is essential for the whole treatment process. There are three main stages of the process being focused on, including the blocking of TXA_2_ formation, the P2Y_12_ ADP receptor and the IIbIIIa integrin. Second, aspirin is also an important anticoagulant drug. It contains acetylsalicylic acid (ASA) which can irreversibly block COX-1 and COX-2 through acetylation of the active sites. Therefore, the production of thromboxane and prostaglandin (PGs) from platelets-membrane arachidonate can be blocked. The inhibition of COX-1 can decrease the formation of prostaglandin H_2_, a metabolic precursor of TXA_2_, which can activate platelet. Additionally, inhibiting coagulation is also a major process. It can be obtained both directly and indirectly. Direct anticoagulation involves the direct inhibition of thrombin or factor Xa, while indirect anticoagulation requires antithrombin activation which can activate thrombin to react.

In clinical practice, physicians usually face the dilemma of choosing appropriate antithrombotic therapy for patients with AMI concomitant AF. Aspirin and a P2Y_12_ inhibitor as the standard DAPT should be indicated to AMI patients, especially during the acute phase, to prevent recurrent MI and stent thrombosis, and to further reduce major adverse cardiac events (MACE), (46, 47) while anticoagulants (VKAs or NOACs) are recommended in patients with AF with a CHA_2_DS_2_-VASc score greater than 2 for preventing stroke and systemic embolic events, no matter whether it is new-onset or prior existent (48).

Unfortunately, TAT comprising DAPT combined with anticoagulant would increase the incidence of bleeding as reported previously, which was thought to be positively associated with the mortality rate (49–51). Therefore, how to balance the efficacy and safety in AMI-NOAF patients is a significant challenge for the optimal antithrombotic treatment, and several retrospective studies have made suggestions on this topic. Although there are enormous new antithrombotic drugs invented for treatment, how to choose an appropriate treatment for different patients is also a big point to focus.

### Future directions

We included 3 studies in our narrative review to explore the efficacy and safety of antithrombotic therapy in AMI patients following new-onset AF. The data of these studies was well collected which increases the credibility and quality of this narrative review. According to the previous studies, we can obviously conclude that antithrombotic therapy plays a significant role in preventing thrombosis formation and reducing the mortality rate in AMI-NOAF patients. And it can also improve the prognosis of these patients. However, there are also some limitations of our study. Firstly, the number of studies included is very small. More clinical studies are needed to increase credibility. Secondly, the study performed by Hofer showed that DAT has a good effect on previously existing AF while there is no improvement in the prognosis of NOAF patients. But TAT exhibits better efficacy in NOAF patients than in pre-existing AF patients. However, some randomized controlled trials demonstrated that compared to TAT, DAT can significantly reduce bleeding events. And the use of TAT has a bias. Only patients with low bleeding risk can choose TAT treatment. Thus, how to choose the optimal regimen is still worthy of serious consideration. Finally, the efficacy of OAC is not evaluated in this review. OAC is a novel drug for these patients, however, there are not many clinical studies on OAC in the treatment of AMI-NOAF patients (46). But what is clear is that OAC can reduce the mortality rate.

## Conclusion and further implications

Atrial fibrillation is a common complication of AMI. At present, the understanding of this complication has been gradually deepened. The pathogenesis of NOAF is not completely clear, further clinical or basic experiments will help to further explore the pathogenesis and break through the bottleneck for precision treatment. Finally, due to prolonged hospitalization, high incidence of hospital complications, high mortality, increased difficulty in hospital treatment management and poor long-term prognosis in patients with AMI complicated with NOAF, efforts should be made to identify those high-risk patients who can be monitored during hospitalization and who can benefit from early treatment. There are some studies showing the benefit of antithrombotic therapy such as OAC, TAT, and DAT, which can prevent thrombosis formation and reduce the risk of bleeding under certain conditions. Antithrombotic therapy for AMI-NOAF patients brings a promising future.

## Data availability statement

The original contributions presented in this study are included in the article/Supplementary material, further inquiries can be directed to the corresponding author.

## Author contributions

All authors listed have made a substantial, direct, and intellectual contribution to the work, and approved it for publication.
