# Supplementary material for: Oral anticoagulant therapy for patients with new-onset atrial fibrillation following acute myocardial infarction: A narrative review
Source: Front Cardiovasc Med. 2022 Nov 3;9:1046298. doi: 10.3389/fcvm.2022.1046298 (PMC9669747; doi:10.3389/fcvm.2022.1046298)
Supplement: Supplementary file 1 [file Table_1.DOCX]

**Supplementary Table 1. The search strategies of this meta-analysis**

|  | **Search terms** | **No.** |
| --- | --- | --- |
| **PubMed** | | |
| #1 | atrial fibrillation | 85543 |
| #2 | Acute myocardial fibrillation OR acute coronary syndrome | 89172 |
| #3 | non-vitamin K antagonist oral anticoagulants OR direct oral anticoagulants OR dabigatran OR rivaroxaban OR apixaban OR edoxaban | 14616 |
| #4 | vitamin K antagonists OR warfarin | 30539 |
| #5 | #1 and #2 and #3 and #4 | 143 |
